# Predicting the metabolic cost of walking while wearing explosive ordnance disposal protective clothing

**DOI:** 10.1186/2046-7648-4-S1-A78

**Published:** 2015-09-14

**Authors:** Aaron Bach, David Borg, Joseph Costello, Ian Stewart

**Affiliations:** 1 Institute of Health and Biomedical Innovation, Queensland University of Technology, Brisbane, Australia; 2 Department of Sport and Exercise Science, University of Portsmouth, Portsmouth, UK

## Introduction

The use of improvised explosive devices (IED) is becoming more prevalent in modern warfare, civil unrest and lone wolf terrorism. This has led to a greater role for explosive ordnance disposal (EOD) technicians to neutralise the threat of IED detonations. As such, the inherent risk to EOD technicians requires them to don a heavy (~34 kg) protective ensemble that subsequently increases the metabolic demand of tasks, such as locomotion. Previous research into the metabolic cost of protective clothing has focused primarily on chemical and fire ensembles [1]. Currently little is known about the metabolic cost of EOD protective clothing. The purpose of this investigation was 1) to quantify the metabolic demand when wearing an EOD ensemble at various speeds of locomotion and 2) establish whether the Pandolf predictive formula [2] is appropriate to estimate of EOD energy expenditure.

## Methods

Seven males (mean(SD) 26 (4) years, 1.82 (0.05) m, 83.7 (10.0) kg, 4.3 (0.4 L.min^-1^) completed six treadmill walking trials at 2.5, 4 and 5.5 km.h^-1 ^(1 % grade) while wearing normal athletic clothing (CON) or an EOD-9 military ensemble (EOD) in a randomised order. Steady state oxygen consumption (6^th ^to 8^th ^minute) was measured to determine the metabolic cost. Observed energy expenditure was also compared to those predicted using Pandolf's formula [2].

## Discussion

This study suggests that a strong correlation (*r^2 ^*= 0.97) is present between observed and predicted EOD energy expenditure. Although, due to the constant under-estimation of the predictive formula based on traditional load carriage (*i.e*. backpack) it may be necessary to make adjustments to the predictive formula to account for the variation in load carriage while wearing the EOD ensemble. For example, the distribution of load around the body, potentially changes gait mechanics due to ensemble size/thickness and increased friction of locomotion.

## Conclusion

The largest differences in energy expenditure were seen at the fastest speed and although strong correlations are present between observed and predicted EOD energy expenditure, the formula significantly underestimated the metabolic cost of walking in an EOD ensemble at every speed tested.

**Figure 1:**
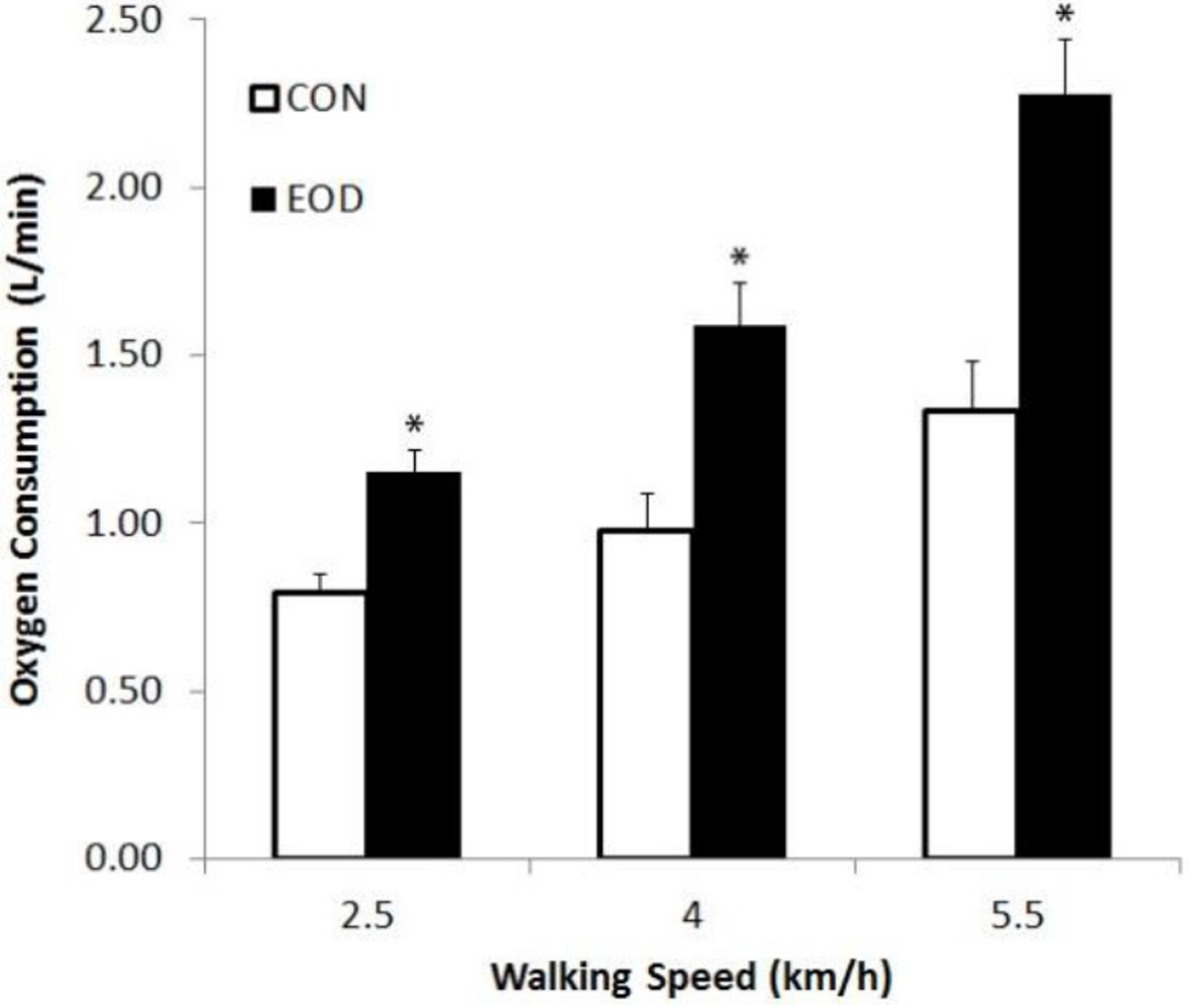
**Oxygen consumption between CON and EOD at different walking speeds**. * significantly different to CON at same speed (p < 0.05).

**Figure 2:**
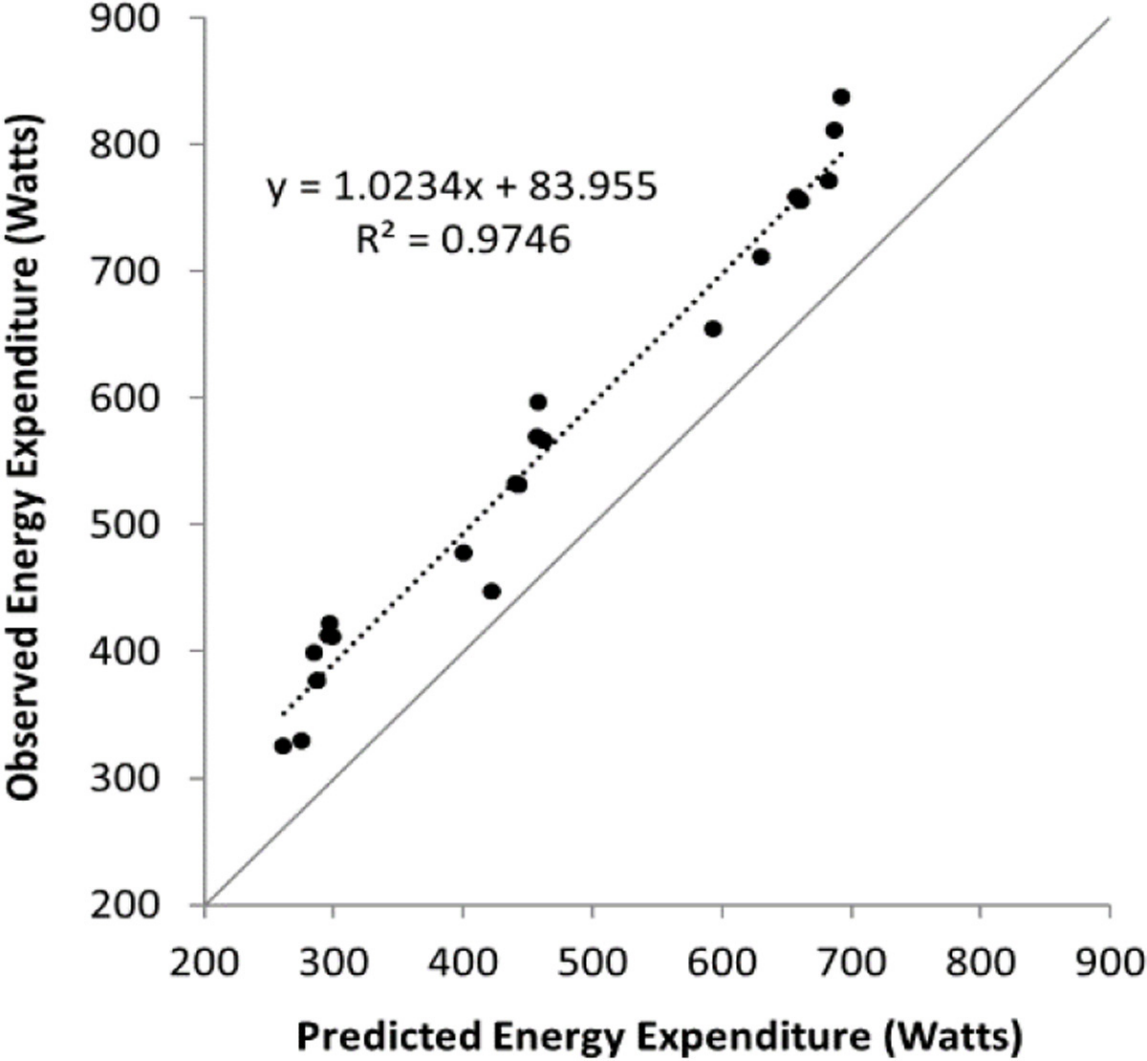
**EOD energy expenditure (W) observed vs. predicted from Pandolf **[1].
